# Fast 4D-PET parametric imaging computation at the whole field of view level: Reliability under simulated conditions of PET KinetiX, a dedicated software solution

**DOI:** 10.1007/s00259-025-07285-0

**Published:** 2025-04-21

**Authors:** Sylvain Faure, Adrien Paillet, Claude Comtat, Florent L. Besson

**Affiliations:** 1 Laboratoire de Mathématique d’Orsay, CNRS/INRIA ParMA Université Paris-Saclay, Orsay, France; 2 CEA/Inserm/CNRS/Université Paris-Saclay, BioMaps, Orsay, France; 3 Department of Nuclear Medicine - Molecular Imaging, Hôpitaux Universitaires Paris-Saclay AP-HP, DMU SMART IMAGING, Hôpital Bicêtre, Le Kremlin-Bicêtre, France; 4 School of Medicine, Université Paris-Saclay, Le Kremlin-Bicêtre, France

**Keywords:** PET, Kinetic modeling, Parametric imaging, Quantification, Simulation, Digital phantom

## Abstract

**Purpose:**

The reliability of a new academic software, PET KinetiX, designed for fast parametric 4D-PET imaging computation, is assessed under simulated conditions.

**Methods:**

4D-PET data were simulated using the XCAT digital phantom and realistic time-activity curves (ground truth). Four hundred analytical simulations were reconstructed using CASToR, an open-source software for tomographic reconstruction, replicating the clinical characteristics of two available PET systems with short and long axial fields of view (SAFOV and LAFOV). A total of 2,800 Patlak and 2TCM kinetic parametric maps of ^18^F-FDG were generated using PET KinetiX. The mean biases and standard deviations of the kinetic parametric maps were computed for each tissue label and compared to the biases of unprocessed SUV data. Additionally, the mean absolute ratio of kinetic-to-SUV contrast-to-noise ratio (CNR) was estimated for each tissue structure, along with the corresponding standard deviations.

**Results:**

The K_i_ and v_b_ parametric maps produced by PET KinetiX faithfully reproduced the predefined multi-tissue structures of the XCAT digital phantom for both Patlak and 2TCM models. Image definition was influenced by the 4D-PET input data: a higher number of iterations resulted in sharper rendering and higher standard deviations in PET signal characteristics. Biases relative to the ground truth varied across tissue structures and hardware configurations, similarly to unprocessed SUV data. In most tissue structures, Patlak kinetic-to-SUV CNR ratios exceeded 1 for both SAFOV and LAFOV configurations. The highest kinetic-to-SUV CNR ratio was observed in 2TCM k₃ maps within tumor regions.

**Conclusion:**

PET KinetiX currently generates K_i_ and v_b_ parametric maps that are qualitatively comparable to unprocessed SUV data, with improved CNR in most cases. The 2TCM k₃ parametric maps for tumor structures exhibited the highest CNR enhancement, warranting further evaluation across different anatomical regions and radiotracer applications.

**Supplementary Information:**

The online version contains supplementary material available at 10.1007/s00259-025-07285-0.

## Introduction

Initially developed in the late 1970 s for brain research, positron emission tomography (PET) has progressively expanded into various fields of clinical practice over the past 25 years [1–6]. Continuous advancements in hardware have led to substantial dose reductions and faster acquisition protocols, facilitating its use across all age groups, including the most vulnerable populations [7–10]. To meet the growing demands of precision medicine, numerous diagnostic and prognostic PET imaging biomarkers have been proposed over the past 15 years [11–16]. Whether derived from advanced statistical methodologies or artificial intelligence-based processing, PET biomarkers are predominantly extracted from static acquisition schemes, which remain the clinical standard worldwide. However, despite increasingly sophisticated post-processing pipelines, static PET metrics remain inherently limited by their semi-quantitative nature. In contrast, since the advent of PET imaging, dynamic acquisition schemes have enabled advanced kinetic modeling of radiotracer behavior, which remains the gold standard for absolute quantification in research [17]. By providing unique biological insights into disease mechanisms, kinetic PET metrics have demonstrated superior diagnostic and prognostic capabilities compared to static PET metrics across various pathologies [18–21]. Until recently, the widespread adoption of kinetic modeling in clinical settings has been hindered by the short axial field of view (SAFOV, typically < 35 cm [22]), of conventional PET systems, along with practical limitations such as prolonged processing times and the need for complex multibed multipass acquisition schemes for whole-body dynamic analyses. The emergence of a new generation of PET systems with a long axial field of view (LAFOV, > 100 cm [22]). has introduced a paradigm shift in PET imaging. These systems offer extended axial coverage and enhanced detection sensitivity, significantly mitigating noise-related challenges. Crucially, they enable true whole-body dynamic acquisitions, paving the way for ultra-high-definition PET imaging in routine clinical practice [23, 24]. In this evolving context, there is growing interest in dynamic PET imaging and voxel-wise kinetic modeling at the whole-FOV level. Large-scale clinical research is essential to validate the relevance of 4D-PET parametric imaging in precision medicine and accelerate its clinical integration. Despite global enthusiasm, the widespread adoption of 4D-PET parametric imaging remains challenging, even in research-oriented PET centers. One critical barrier is the availability of universal, fast, and efficient dedicated software. To address this, we recently developed PET KinetiX, a clinically oriented software solution designed for rapid parametric imaging across the entire FOV for any reconstructed 4D-PET dataset (single or multipass), regardless of PET system type (SAFOV or LAFOV) [25]. We have previously demonstrated the time efficiency of PET KinetiX, validated its clinical performance against the reference research standard (PMOD-PKIN version 4.4), and assessed its operability across various PET systems, including the Signa PET/MR (GE Healthcare), Biograph mCT Flow (Siemens Healthineers), Biograph Vision 600 (Siemens Healthineers), and Vision Quadra (Siemens Healthineers) [25].

In recent years, PET system manufacturers have progressively introduced kinetic modeling solutions for clinical research applications. While commendable, these solutions remain manufacturer-dependent and are often limited to simplified kinetic models. Moreover, their development is constrained by market-driven priorities and hardware renewal cycles. In this context, PET KinetiX represents a significant advancement in 4D-PET parametric imaging, offering simplicity, universality, and the flexibility to integrate state-of-the-art methodologies. As such, it has the potential to facilitate large-scale, multicenter studies and support the clinical validation of kinetic modeling. Before its broader clinical deployment, the reliability of PET KinetiX must first be demonstrated under rigorously controlled conditions. The present study aims to evaluate the reliability of PET KinetiX under simulated conditions where the ground truth is known.

## Methods

### Phantom simulations

**Fig. 1:**
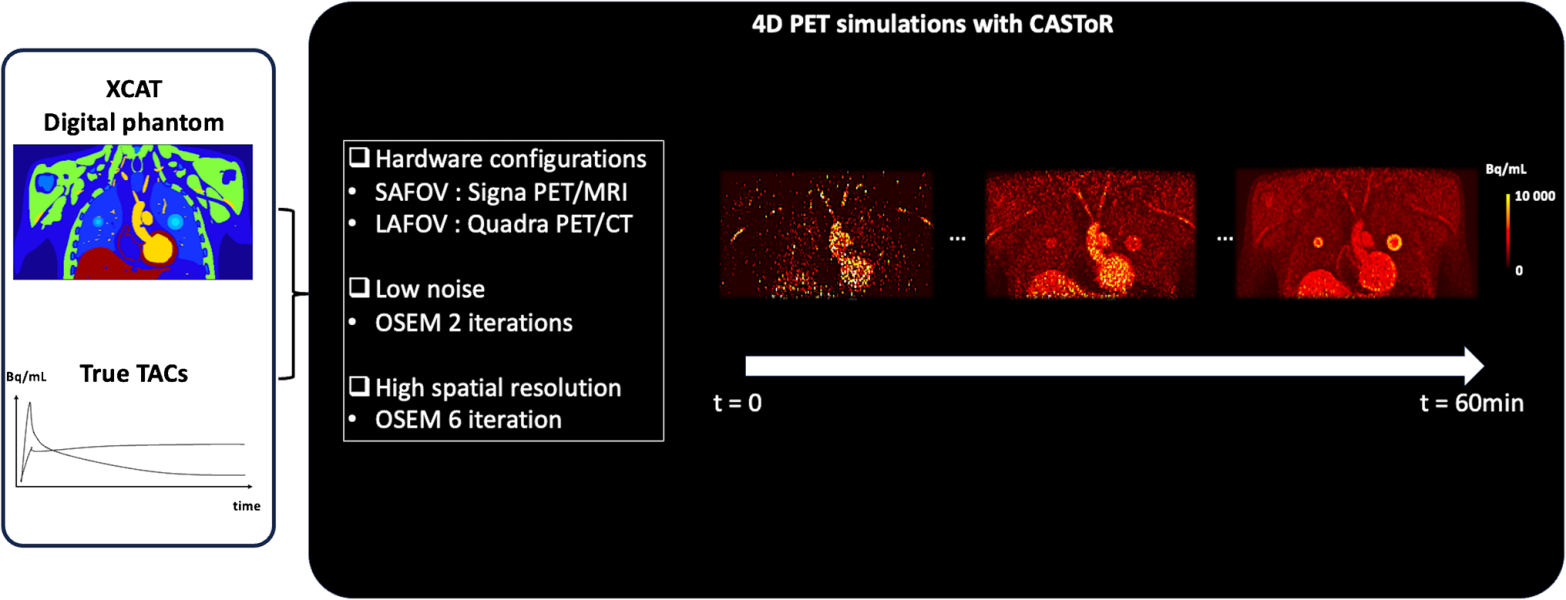
Simulation process. XCAT digital phantom and true TACs were used to simulate realistic 4D-PET data with the Customizable and Advanced Software for Tomographic Reconstruction (CASToR). For this purpose, SAFOV and LAFOV-like PET data were simulated with low noise (OSEM algorithm with 2 iterations) and high spatial resolution (OSEM algorithm with 6 iterations)

A general overview of the simulation process is provided in Fig. 1. For this purpose, the XCAT anthropomorphic digital phantom (Duke University, Durham, North Carolina, USA) and realistic time-activity curves (TACs) of ^18^F-FDG were used to generate synthetic thoracic 4D-PET data. The digital phantom offers a highly detailed model of human anatomy (voxel size: 1 mm × 1 mm × 1 mm), enabling the virtual modeling of multiple tissue classes [26].

True TACs were extracted from one-hour dynamic thoracic PET data acquired in non-small cell lung cancer patients using a SAFOV PET/MRI scanner (Signa PET/MR, GE Healthcare, Waukesha, WI, USA, AFOV = 25 cm). These data had been previously analyzed in [27] and [28]. The estimated kinetic parameters (K₁, k₂, k₃, and v_b_) of these TACs reflect the real-life kinetic behavior of ^18^F-FDG and were used as templates to simulate realistic TACs (ground truth) for eight predefined reference tissue structures: lung tumors **(**center and border), muscles, fat, bone, heart, lungs, liver, and spleen (Table 1). Each temporal frame of the dynamic phantom was numerically forward-projected into the sinogram domain of the simulated scanner, accounting for photon attenuation. Pseudo-random Poisson noise was added to the ground-truth sinogram 100 times, generating 100 independent noisy realizations of the same ground-truth phantom. Additionally, random coincidences were simulated. The total number of true and random coincidences was determined based on real patient data. For each temporal frame, both the noise-free ground-truth sinogram and the 100 noisy sinograms were reconstructed using the OSEM algorithm (with 2 iterations for low-noise reconstructions and 6 iterations for high spatial resolution reconstructions, without point spread function modeling or post-filtering). Two scanner configurations were simulated:

- **SAFOV-like configuration**, based on the specifications of the Signa PET/MR scanner [29]. The total numbers of true and random coincidences were set according to patient data.PET time-of-flight was not simulated. The number of OSEM subsets (N = 28) and the reconstructed voxel size (2.34 mm × 2.34 mm × 2.78 mm) were identical to clinical standard practice.
- **LAFOV-like configuration**, based on the characteristics of the PET/CT Vision Quadra scanner (Siemens Healthineers, Erlangen, Germany) [30]. The total numbers of true and random coincidences in the thoracic region were multiplied by a factor of 3.5, accounting for the sensitivity difference between the Signa PET/MR and the central axial FOV of the Vision Quadra scanner operated in 322 maximum ring difference mode. PET time-of-flight was simulated with a 214-picosecond resolution. The number of OSEM subsets (N = 19) and the reconstructed voxel size (1.65 mm × 1.65 mm × 1.645 mm) were identical to clinical standard practice.

**Table 1:** Reference values for kinetic parameters used for simulations

Structures	2 TCM					Patlak	
	K_1_ (mL/min/cm^3^)	k_2_ (min^−1^)	k_3_ (min^−1^)	v_b_ (dimensionless)	Ki (mL/min/cm^3^)	Ki (mL/min/cm^3^)	v_b_ (dimensionless)
Tumors							
Border	0.08	0.18	0.08	0.07	0.03	0.02	0.22
Center	0.05	0.19	0.06	0.05	0.01	0.01	0.17
Liver	0.10	0.34	0.04	0.33	0.011	0.007	0.468
Lungs	0.017	0.30	0.027	0.067	0.001	0.001	0.106
Bone	0.037	0.22	0.044	0.11	0.006	0.005	0.201
Heart	0.11	0.38	0.066	0.17	0.016	0.013	0.30
Spleen	0.055	0.28	0.030	0.23	0.005	0.004	0.34
Muscle	0.038	0.24	0.018	0.007	0.003	0.003	0.15
Soft-tissue Fat	0.015	0.29	0.024	0.011	0.001	0.001	0.05

For 2 TCM and Patlak parameters, these values were estimated from real-life one-hour TACs acquired previously on SAFOV hybrid PET/MRI system for thoracic oncology purpose [27, 28]

The forward projection of the digital phantom, noise addition, and image reconstruction were performed using the Customizable and Advanced Software for Tomographic Reconstruction** (**CASToR**)** platform [31]).

For all thoracic simulations, dynamic PET data were histogrammed into multiframe sinograms to match the true TACs: 41 frames consisting of 12 × 10 s, 12 × 20 s, 4 × 60 s, 5 × 120 s, and 8 × 300 s, respectively. Random coincidences, attenuation, decay, and time-of-flight (for the LAFOV-like configuration) were accounted for in both simulations and reconstructions. Due to differences in reconstructed voxel sizes, the matrix dimensions for the thoracic region differed between configurations (SAFOV: 256 × 256 × 89 and LAFOV: 380 × 380 × 159) as well as across voxel-wise analyses (see Table 2). Finally, SUV PET maps were computed from the last frame of each simulated reconstruction using Eq. (1):

1 $$SUV=Activity/\left(injected\;dose\times\right.2^{(-\lambda t)})\times Weight$$

where λ is the decay constant $\lambda = \frac{Ln2}{T}$ with T = 110 min, the half-life of ^18^F-FDG, and *t* representing the delay between injection and image acquisition.

**Table 2:** Number of voxels per structure, according to each configuration characteristics

Structures	XCAT phantom	PET configuration	
		SAFOV-like	LAFOV-like
Tumor R			
Border	3 654 voxels	191 voxels	602 voxels
Center	515 voxels	37 voxels	96 voxels
Tumor L			
Border	12 356 voxels	677 voxels	2 225 voxels
Center	1 791 voxels	104 voxels	307 voxels
Liver	583 052 voxels	34 835 voxels	118 328 voxels
Lungs	1 154 991 voxels	71 187 voxels	238 064 voxels
Bone	752 945 voxels	36 431 voxels	124 149 voxels
Heart	161 633 voxels	6 817 voxels	22 858 voxels
Spleen	16 130 voxels	809 voxels	2 740 voxels
Muscle	3 962 442 voxels	231 037 voxels	782 435 voxels
Soft-tissue Fat	6 889 690 voxels	366 571 voxels	1 252 929 voxels

To ensure consistency across all simulations, a standardized body weight of 60 kg and an injected dose of 4 MBq/kg of ^18^F-FDG were systematically applied.

### Parametric image processing and analysis

**Fig. 2:**
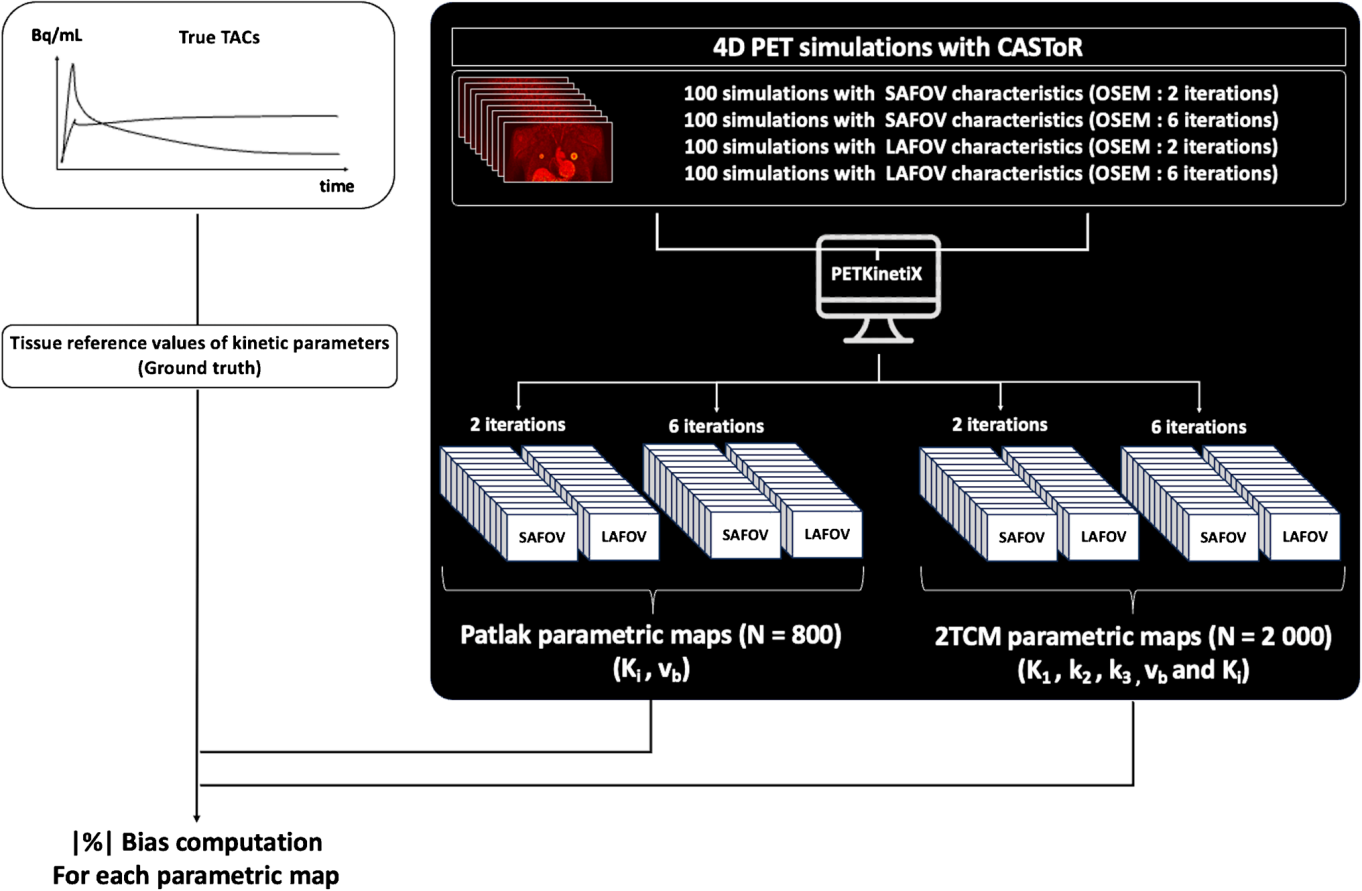
Data analyses. The 400 simulated 4D-PET data were processed individually with PET KinetiX to generate a total of 2 800 parametric maps of kinetic parameters at the whole FOV-level: 800 Patlak maps (400 K_i_ and v_b_ maps respectively) and 2 000 2 TCM maps (400 K_1_, k_2_, k_3_, v_b_ and K_i_ maps respectively)

Image processing and analysis are summarized in Fig. 2. All reconstructed 4D-PET data were processed using PET KinetiX [25] on a MacBook Pro – Apple M1 Max with 64 GB of RAM. Parametric maps were computed using an indirect method with an image-derived input function defined on the thoracic aorta. These maps provided voxel-wise kinetic parameters according to both the simplified Patlak model (K_i_ and v_b_) and the irreversible two-tissue compartment model (2 TCM, yielding the following parameters: K_1_, k_2_, k_3_, v_b_, and K_i_, where K_i_ = K_1_ $\times$ k_3_/[k_2_ + k_3_]).

For each of the four configurations (2 and 6 OSEM iterations, SAFOV-like and LAFOV-like scanners), the voxel-wise mean and standard deviation of the parametric maps were computed across the 100 noise realizations. The mean bias of PET KinetiX per tissue label was then calculated according to Eq. (2):

2 $$\text{Bias }(\text{\%})=\left|\left(<\text{PET KinetiX}>-\text{ ground truth}\right)/\text{ ground truth}\right|\times 100$$

where 〈PET KinetiX〉 represents the mean value across the 100 noise realizations. The associated standard deviations were also reported.

For consistency in comparison, biases were also computed for the corresponding SUV data, using Eq. (3):

3 $$\text{Bias }(\text{\%})=|(<\text{Noisy SUV SAFOV or LAFOV}>-\text{ ground truth}) /\text{ ground truth }|\times 100$$

where the ground truth corresponds to noise-free data, and 〈Noisy SUV_SAFOV or LAFOV_〉represents the mean across the 100 noise realizations. The associated standard deviations were also reported.

The percentage normalized bias (NBias) and normalized standard deviation (NSD) were estimated for all 11 tissue structures as a function of the number of iterations (2 or 6). NBias and NSD were computed over 100 simulations and plotted together to generate noise-bias trade-off lines for each tissue label and scanner configuration (SAFOV or LAFOV-like).

For each mean parametric map, the absolute contrast-to-noise ratio (CNR) was computed for each tissue label, using the superior vena cava (venous blood pool) as background reference, following Eq. (4):

4 $$CNR|=\left|\frac{{(ROI}_{tissue}-{ROI}_{Bck})}{STD \left({ROI}_{Bck}\right)}\right|,$$

Here ROI_tissue_ and ROI_Bck_ represent small regions of interest defined on the tissue target and the background signal, respectively, and STD (ROI_Bck_) denotes the standard deviation of the background signal within the mean of all parametric maps.

Finally, the mean absolute kinetic-to-SUV CNR ratio was computed for each tissue structure, along with the corresponding standard deviations**.**

## Results

### Visual quality rendering

**Fig. 3:**
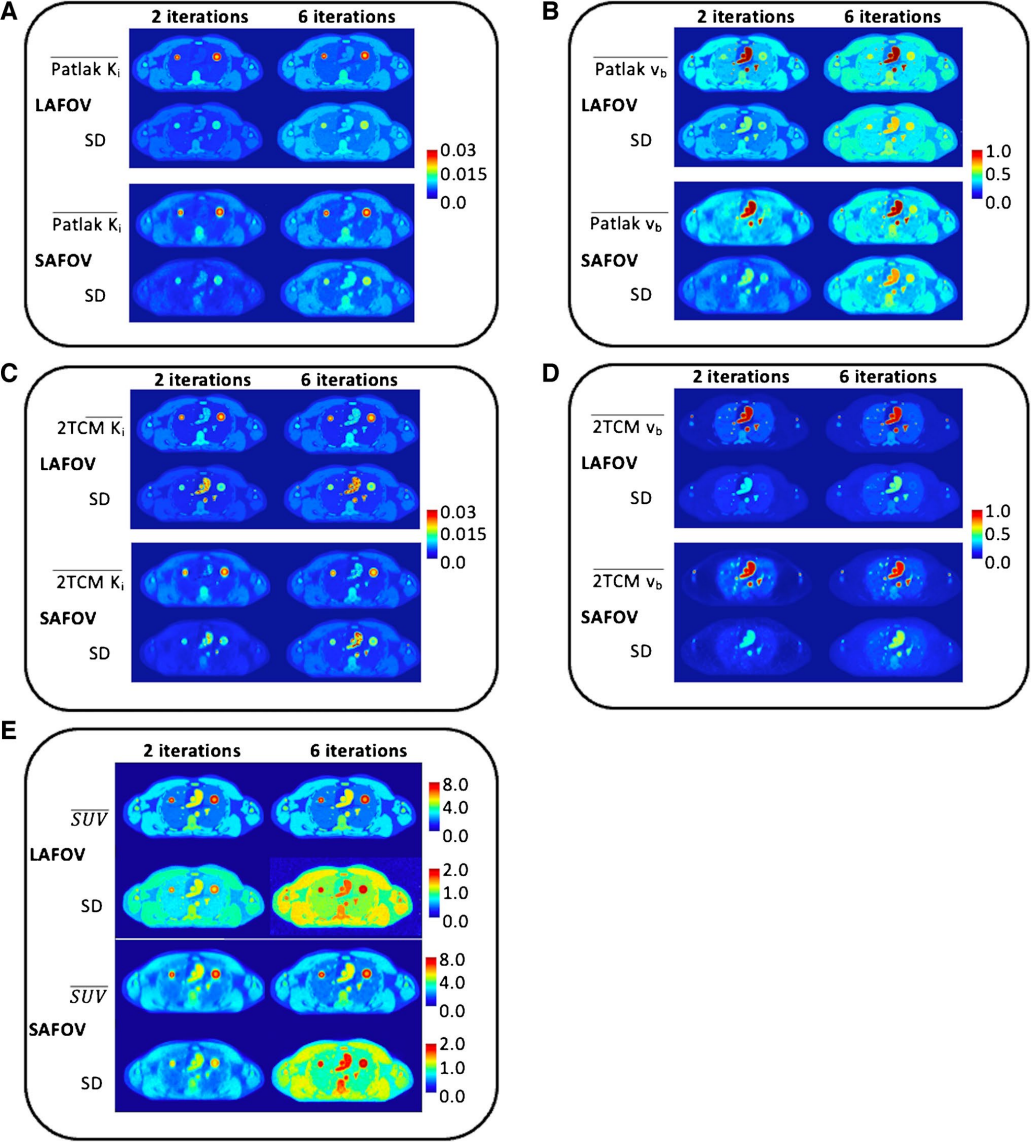
Mean and SD of parametric maps. **A**, **B**, **C**, **D**: For each configuration, $\overline{X }$ and SD correspond respectively to the mean and standard deviations of the parameters estimated from the 100 simulations with PET KinetiX. **A**. Patlak Ki; **B**: Patlak v_b_; **C**: 2 TCM Ki; **D**: 2 TCM v_b_. And **E**: mean and standard deviations of the SUV variabilities estimated from the 100 simulations

Illustrations of the mean and standard deviation (SD) parametric maps computed from the simulated data using PET KinetiX (Patlak and 2 TCM) are shown in Fig. 3, alongside the variability of the corresponding simulated SUV data. The K_i_ and v_b_ maps exhibited excellent visual quality, ensuring high structural consistency across tissue regions (Fig. 3A-D) compared to SUV data (Fig. 3E). Overall, the LAFOV-like configuration provided sharper definition in both kinetic and SUV parametric maps compared to the SAFOV-like configuration (Fig. 3A-E). As expected, for both SAFOV and LAFOV-like configurations, increasing the number of iterations enhanced the sharpness of both kinetic and SUV data, albeit at the cost of increased noise levels (Fig. 3A-E). In kinetic parametric maps, vascular structures were particularly well-defined in v_b_ images compared to Ki images (Fig. 3B and 3D), whereas in SUV data, vascular and non-vascular structures appeared blended together (Fig. 3E).

### Quantitative biases of PET KinetiX under realistic conditions (noisy data)

The overall biases of PET KinetiX are summarized in Tables 3 and 4. Compared to the SAFOV-like configuration, the LAFOV-like configuration demonstrated a median bias reduction of:

 − 37% for 2 TCM and − 56% for Patlak kinetic parameters at 2 iterations (low noise).
 − 27% for 2 TCM and − 3% for Patlak kinetic parameters at 6 iterations (high spatial resolution).

**Table 3:** Biases (in %) of PET KinetiX for 2 iterations, no post filtering

Configuration	Structures	2 TCM					Patlak	
		K_1_ (in %)	k_2_ (in %)	k_3_ (in %)	v_b_ (in %)	K_i_ (in %)	K_i_ (in %)	v_b_ (in %)
SAFOV	Tumor R							
	Border	42.8 $\pm$ 39.2	13.0 $\pm$ 9.7	14.1 $\pm$ 9.3	17.1 $\pm$ 14.3	41.9 $\pm$ 4.6	29.7 $\pm$ 9.3	27.7 $\pm$ 25.0
	Center	20.5 $\pm$ 39.7	23.2 $\pm$ 17.9	24.4 $\pm$ 14.4	77.4 $\pm$ 35.5	34.6 $\pm$ 6.0	21.8 $\pm$ 15.9	20.0 $\pm$ 28.6
	Tumor L							
	Border	30.3 $\pm$ 53.8	13.4 $\pm$ 7.7	6.3 $\pm$ 7.7	22.0 $\pm$ 16.1	31.3 $\pm$ 2.9	17.8 $\pm$ 14.7	16.8 $\pm$ 31.2
	Center	22.2 $\pm$ 81.3	22.0 $\pm$ 14.6	10.1 $\pm$ 9.2	14.1 $\pm$ 19.5	3.9 $\pm$ 8.1	18.6 $\pm$ 27.5	15.7 $\pm$ 32.2
	Liver	9.1 $\pm$ 23.0	37.5 $\pm$ 3.0	52.4 $\pm$ 3.5	24.0 $\pm$ 7.0	44.4 $\pm$ 3.2	17.0 $\pm$ 30.1	12.4 $\pm$ 16.8
	Lungs	92.1 $\pm$ 162.7	4.8 $\pm$ 8.8	24.1 $\pm$ 7.5	11.5 $\pm$ 12.2	5.2 $\pm$ 3.7	27.3 $\pm$ 40.3	17.7 $\pm$ 17.8
	Bone	26.0 $\pm$ 71.4	7.1 $\pm$ 8.7	27.2 $\pm$ 5.7	47.6 $\pm$ 1.9	44.9 $\pm$ 2.7	25.6 $\pm$ 13.5	27.1 $\pm$ 9.3
	Heart	30.9 $\pm$ 34.6	38.7 $\pm$ 4.9	38.8 $\pm$ 4.1	5.2 $\pm$ 14.7	41.4 $\pm$ 1.8	26.6 $\pm$ 13.4	6.4 $\pm$ 20.8
	Spleen	26.9 $\pm$ 30.6	46.8 $\pm$ 5.6	59.0 $\pm$ 4.6	61.4 $\pm$ 5.7	61.4 $\pm$ 6.1	8.6 $\pm$ 21.0	18.3 $\pm$ 9.7
	Muscle	9.4 $\pm$ 55.4	9.8 $\pm$ 5.2	6.8 $\pm$ 8.3	88.2 $\pm$ 91.7	23.9 $\pm$ 2.9	6.9 $\pm$ 26.1	6.4 $\pm$ 14.7
	Soft-tissue Fat	43.8 $\pm$ 82.8	12.5 $\pm$ 2.6	12.8 $\pm$ 6.8	55.8 $\pm$ 42.6	2.5 $\pm$ 2.1	32.7 $\pm$ 34.6	47.7 $\pm$ 24.2
LAFOV	Tumor R							
	Border	4.6 $\pm$ 2.7	5.8 $\pm$ 3.9	24.8 $\pm$ 3.1	9.3 $\pm$ 4.5	24.3 $\pm$ 1.9	6.9 $\pm$ 1.5	26.5 $\pm$ 7.0
	Center	9.9 $\pm$ 7.9	16.1 $\pm$ 11.6	25.9 $\pm$ 8.4	12.9 $\pm$ 9.0	24.8 $\pm$ 4.5	8.9 $\pm$ 4.2	18.0 $\pm$ 12.6
	Tumor L							
	Border	1.9 $\pm$ 2.6	6.5 $\pm$ 2.8	22.2 $\pm$ 1.8	10.9 $\pm$ 2.9	20.7 $\pm$ 1.5	3.3 $\pm$ 1.2	29.2 $\pm$ 6.2
	Center	16.9 $\pm$ 6.3	12.0 $\pm$ 6.2	16.5 $\pm$ 4.6	7.3 $\pm$ 6.2	13.8 $\pm$ 2.4	4.9 $\pm$ 2.9	26.5 $\pm$ 8.8
	Liver	11.3 $\pm$ 5.3	16.2 $\pm$ 3.0	25.1 $\pm$ 2.2	7.7 $\pm$ 1.9	21.7 $\pm$ 2.3	2.4 $\pm$ 1.9	2.6 $\pm$ 2.8
	Lungs	47.6 $\pm$ 4.3	20.7 $\pm$ 1.6	31.6 $\pm$ 1.5	10.5 $\pm$ 1.5	24.8 $\pm$ 1.6	15.8 $\pm$ 2.6	10.9 $\pm$ 3.4
	Bone	22.8 $\pm$ 3.4	2.1 $\pm$ 1.5	30.6 $\pm$ 1.4	19.1 $\pm$ 2.4	26.0 $\pm$ 1.5	4.2 $\pm$ 1.5	4.4 $\pm$ 3.3
	Heart	15.5 $\pm$ 2.3	30.1 $\pm$ 1.7	24.6 $\pm$ 1.5	6.0 $\pm$ 2.3	20.5 $\pm$ 1.9	6.5 $\pm$ 1.2	4.6 $\pm$ 3.4
	Spleen	20.3 $\pm$ 5.4	10.3 $\pm$ 3.4	28.2 $\pm$ 3.1	12.4 $\pm$ 1.9	29.3 $\pm$ 2.2	3.4 $\pm$ 2.3	2.6 $\pm$ 2.5
	Muscle	6.1 $\pm$ 2.5	8.0 $\pm$ 1.0	7.6 $\pm$ 1.9	63.1 $\pm$ 6.2	18.4 $\pm$ 1.5	3.3 $\pm$ 2.3	7.7 $\pm$ 3.4
	Soft-tissue Fat	15.2 $\pm$ 2.7	16.6 $\pm$ 0.7	6.0 $\pm$ 1.3	6.8 $\pm$ 3.5	14.8 $\pm$ 1.5	16.6 $\pm$ 2.3	29.5 $\pm$ 4.0

For each parameter, the biases (in %) are expressed as mean $\pm$ Standard Deviation estimated over 100 replicates

**Table 4:** Biases (in %) of PET KinetiX for 6 iterations, no post filtering

Configuration	Structures	2 TCM					Patlak	
		K_1_ (in %)	k_2_ (in %)	k_3_ (in %)	v_b_ (in %)	K_i_ (in %)	K_i_ (in %)	v_b_ (in %)
SAFOV	Tumor R							
	Border	29.1 $\pm$ 4.4	19.4 $\pm$ 8.0	48.6 $\pm$ 5.0	7.8 $\pm$ 5.2	44.2 $\pm$ 2.1	23.3 $\pm$ 2.4	32.6 $\pm$ 12.3
	Center	20.6 $\pm$ 16.8	20.1 $\pm$ 12.4	50.6 $\pm$ 12.9	86.5 $\pm$ 31.7	39.3 $\pm$ 7.2	15.1 $\pm$ 7.9	55.6 $\pm$ 28.9
	Tumor L							
	Border	19.4 $\pm$ 3.2	20.4 $\pm$ 4.6	43.2 $\pm$ 3.7	5.1 $\pm$ 4.1	34.6 $\pm$ 1.8	11.3 $\pm$ 2.0	43.0 $\pm$ 10.4
	Center	12.0 $\pm$ 9.1	18.1 $\pm$ 10.2	35.1 $\pm$ 7.6	21.1 $\pm$ 13.5	13.3 $\pm$ 4.8	15.9 $\pm$ 6.3	62.5 $\pm$ 18.4
	Liver	5.0 $\pm$ 3.4	51.5 $\pm$ 1.8	70.4 $\pm$ 1.3	34.4 $\pm$ 2.3	54.4 $\pm$ 1.3	30.1 $\pm$ 2.1	24.0 $\pm$ 6.7
	Lungs	61.1 $\pm$ 6.8	29.2 $\pm$ 2.7	38.9 $\pm$ 2.6	14.9 $\pm$ 3.1	19.7 $\pm$ 3.0	42.0 $\pm$ 3.5	24.9 $\pm$ 7.1
	Bone	5.5 $\pm$ 3.1	28.2 $\pm$ 2.3	55.9 $\pm$ 1.5	36.8 $\pm$ 2.3	47.0 $\pm$ 1.2	5.1 $\pm$ 1.5	19.0 $\pm$ 6.5
	Heart	20.5 $\pm$ 3.8	55.5 $\pm$ 2.0	64.6 $\pm$ 1.4	3.1 $\pm$ 2.2	46.9 $\pm$ 1.3	9.6 $\pm$ 1.6	50.7 $\pm$ 8.4
	Spleen	62.9 $\pm$ 3.6	78.3 $\pm$ 1.7	85.0 $\pm$ 2.3	80.9 $\pm$ 2.0	81.1 $\pm$ 1.8	43.7 $\pm$ 5.1	53.2 $\pm$ 5.1
	Muscle	15.5 $\pm$ 1.8	21.6 $\pm$ 1.2	3.0 $\pm$ 2.0	160.5 $\pm$ 15.0	31.5 $\pm$ 1.6	11.7 $\pm$ 2.0	15.1 $\pm$ 6.4
	Soft-tissue Fat	13.1 $\pm$ 3.2	45.7 $\pm$ 1.2	40.0 $\pm$ 1.6	69.0 $\pm$ 7.2	22.5 $\pm$ 1.8	39.7 $\pm$ 2.1	63.8 $\pm$ 8.9
LAFOV	Tumor R							
	Border	10.0 $\pm$ 2.6	16.8 $\pm$ 4.5	42.2 $\pm$ 2.8	3.9 $\pm$ 3.0	29.6 $\pm$ 2.5	6.5 $\pm$ 1.7	58.6 $\pm$ 8.4
	Center	8.1 $\pm$ 6.7	14.7 $\pm$ 9.4	43.6 $\pm$ 7.3	9.4 $\pm$ 7.7	31.8 $\pm$ 4.1	6.7 $\pm$ 4.4	51.3 $\pm$ 15.8
	Tumor L							
	Border	6.8 $\pm$ 2.0	17.6 $\pm$ 2.2	40.8 $\pm$ 1.5	2.7 $\pm$ 2.8	26.8 $\pm$ 2.3	2.8 $\pm$ 2.0	66.3 $\pm$ 7.2
	Center	7.0 $\pm$ 5.5	17.2 $\pm$ 5.6	38.3 $\pm$ 4.5	17.9 $\pm$ 7.6	22.5 $\pm$ 3.2	6.8 $\pm$ 4.2	68.8 $\pm$ 10.5
	Liver	17.7 $\pm$ 7.2	29.0 $\pm$ 2.1	45.3 $\pm$ 1.3	10.6 $\pm$ 1.9	32.6 $\pm$ 2.8	9.6 $\pm$ 3.3	10.1 $\pm$ 3.9
	Lungs	50.1 $\pm$ 5.6	32.6 $\pm$ 1.1	43.1 $\pm$ 1.1	9.9 $\pm$ 1.5	22.4 $\pm$ 2.4	57.5 $\pm$ 4.2	36.9 $\pm$ 4.7
	Bone	20.2 $\pm$ 4.5	17.6 $\pm$ 1.5	45.3 $\pm$ 0.9	18.9 $\pm$ 2.5	30.9 $\pm$ 2.0	6.3 $\pm$ 2.9	28.1 $\pm$ 4.5
	Heart	19.3 $\pm$ 2.9	46.8 $\pm$ 1.2	47.0 $\pm$ 1.0	7.5 $\pm$ 1.9	28.5 $\pm$ 2.3	3.4 $\pm$ 1.8	26.4 $\pm$ 4.7
	Spleen	32.7 $\pm$ 7.0	18.3 $\pm$ 2.6	40.4 $\pm$ 2.6	18.5 $\pm$ 2.5	32.2 $\pm$ 2.9	16.2 $\pm$ 3.8	9.0 $\pm$ 3.8
	Muscle	4.1 $\pm$ 2.0	11.1 $\pm$ 0.7	11.3 $\pm$ 1.4	128.0 $\pm$ 8.0	21.2 $\pm$ 2.1	25.4 $\pm$ 3.3	31.5 $\pm$ 4.6
	Soft-tissue Fat	1.4 $\pm$ 3.1	39.9 $\pm$ 0.6	32.7 $\pm$ 0.8	43.7 $\pm$ 4.5	24.6 $\pm$ 2.1	41.7 $\pm$ 3.7	65.3 $\pm$ 5.6

For each parameter, the biases (in %) are expressed as mean $\pm$ Standard Deviation estimated over 100 replicates

**Fig. 4:**
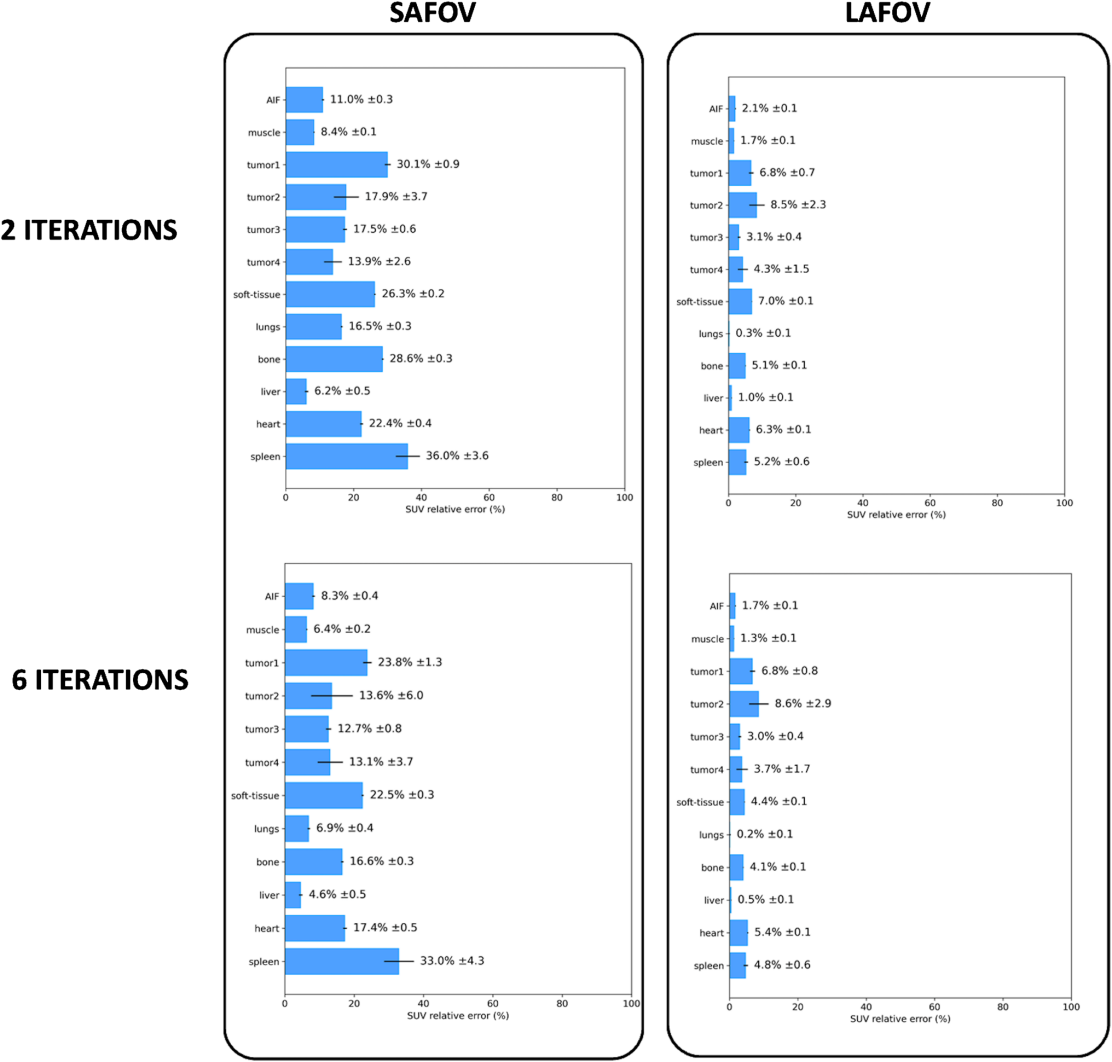
SUV biases. The bias of SUV within the arterial region of interest we used for image derived input function (AIF) are also provided here

Notably, the intrinsic biases of unprocessed SUV data ranged from:

- 6.2 ± 0.5% to 36.0 ± 3.6% (2 iterations) and 4.6 ± 0.5% to 33.0 ± 4.3% (6 iterations) for the SAFOV-like configuration.
- 0.3 ± 0.1% to 8.5 ± 2.3% (2 iterations) and 0.2 ± 0.1% to 8.6 ± 2.9% (6 iterations) for the LAFOV-like configuration (Fig. 4).

**Fig. 5:**
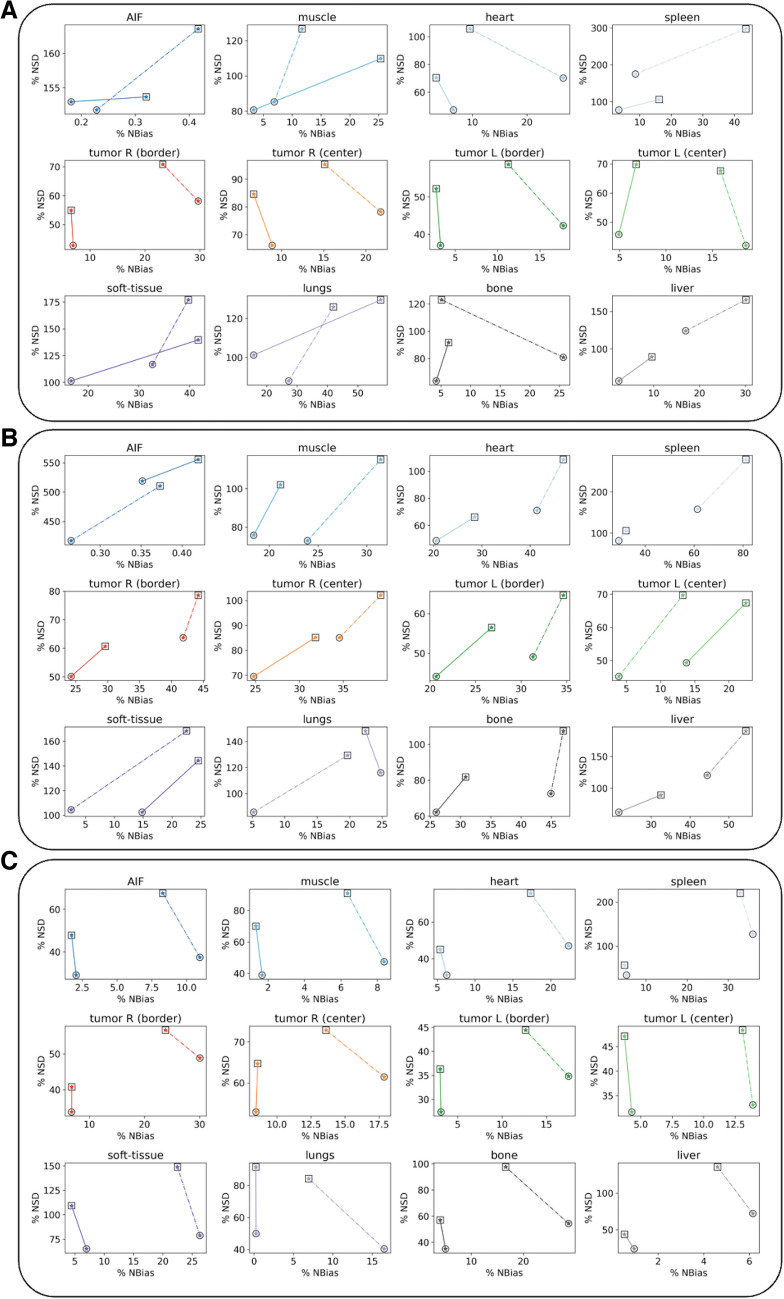
Quantitative noise-bias trade-off plots. Quantitative noise-bias trade-off plots on the 11 tissue structures over 100 noise replicates, for 2 and 6 iterations (circle and square markers respectively). For each analysis, SAFOV (dotted lines) and LAFOV (continuous lines)-configurations are compared. **A**. K_i_ (Patlak); **B**. K_i_ (2 TCM); and **C**. SUV. In all the cases, the dashed curve represents SAFOV, while the continuous curve represents LAFOV

The noise-bias trade-off plots further emphasized the superiority of the LAFOV-PET configuration over SAFOV-PET (Fig. 5).

**Fig. 6:**
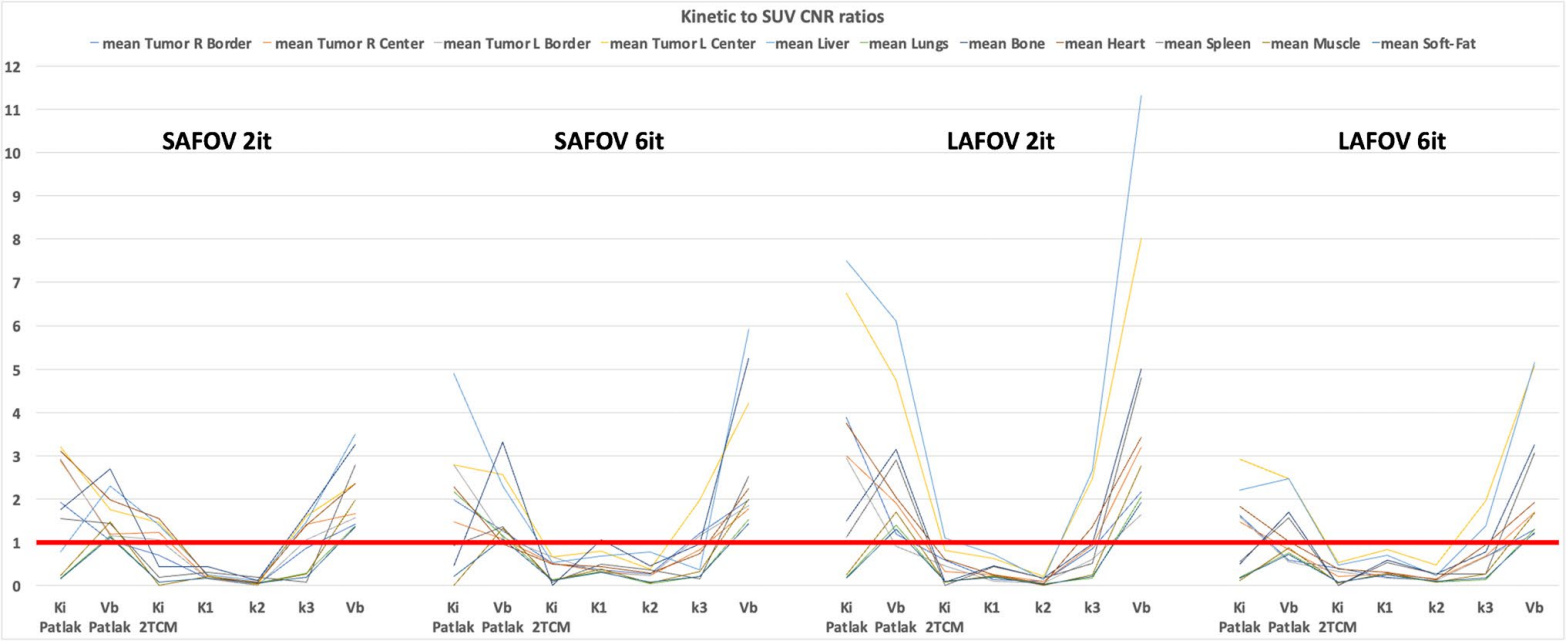
Kinetic to SUV CNR ratios. The red line corresponds to kinetic to SUV CNR ratio of 1. All the results above this line correspond to CNR_kinetic_ higher than CNR_SUV_, whereas all the results under this line correspond to CNR_kinetic_ lower than CNR_SUV_

Regardless of the configuration, the mean absolute kinetic-to-SUV CNR ratio was significantly improved using Patlak parametric maps (both K_i_ and v_b_) across most tissue structures. Also, the highest ratio was observed in 2 TCM k_3_ maps of tumors (Fig. 6).

## Discussion

In this digital PET phantom study, we evaluated under simulated conditions the reliability of PET KinetiX, an academic software designed for fast indirect parametric 4D-PET imaging at the whole field-of-view (FOV) level [25]. We simulated 400 4D-PET datasets of the same thorax model (with 11 tissue labels, including 2 lung tumors) and processed them with PET KinetiX, generating 2,800 realistic 3D kinetic parametric maps of ^18^F-FDG (Patlak: 800 whole-FOV maps; 2 TCM: 2,000 whole-FOV maps).

The K_i_ and v_b_ parametric maps generated with PET KinetiX faithfully reproduced the predefined multi-tissue structures of the XCAT digital phantom, for both Patlak and 2 TCM models. As expected, the image definition of these parametric maps depended on the noise characteristics of the 4D-PET input data: more iterations led to sharper tissue structures, but also increased noise in PET signal characteristics. The LAFOV PET configuration produced higher quality parametric maps than the SAFOV configuration, particularly for small structures (e.g., at tissue interfaces and within the two simulated lung tumors). Regarding 2 TCM modeling, while K_1_ parametric maps showed similar tissue rendering to K_i_ and v_b_ maps, the k_2_ and k_3_ parametric maps appeared less regionally structured (supplementary material 1). However, tumor targets were particularly well-highlighted in k_3_ maps. Overall, the parametric maps generated by PET KinetiX were influenced by reconstruction parameters and noise characteristics, similarly to unprocessed static SUV data. The observed biases for K_i_ maps were comparable to those reported by Karakatsanis et al. [32, 33]. Moreover, the kinetic-to-SUV CNR ratios varied by parameter, with Patlak (K_i_ and v_b_) and 2 TCM v_b_ maps showing improved CNR in most tissue structures (Fig. 6). Conversely, K_1_, k_2_, and k_3_ maps exhibited lower kinetic-to-SUV CNR ratios, with k_2_ maps performing the worst, regardless of the tissue structure analyzed. The amount of noise in a parametric map at the voxel level depends on voxel size. This study demonstrated the reliability of PET KinetiX for voxel sizes identical to clinical standard practice, where high spatial resolution is prioritized for tumor parametric imaging. The higher sensitivity of LAFOV PET enables high-resolution parametric imaging.

A growing number of clinical ^18^F-FDG PET studies have reported a higher target-to-background ratio for K_i_ Patlak parametric maps compared to standard SUV data [20, 34–36], Several clinical applications are currently being explored [20, 35–38]. However, whole-FOV parametric imaging remains challenging due to high computational demands, especially with 4D-PET datasets of high temporal and spatial resolution [39, 40]. Additionally, advanced kinetic modeling is not yet routinely available on most standard workstations, limiting large-scale research into kinetic-based biomarkers. Among these biomarkers:

- K_1_ parameter estimates blood flow in cases of high extraction fraction (e.g., freely diffusible radiotracers or tissues with high permeability-surface products when using non-freely diffusible radiotracers) [41]. In cases of low extraction fraction, K_1_ instead reflects tissue permeability. This could explain the variability in biases observed between high extraction tissues (tumors, liver, spleen, bone) and low extraction tissues (lungs) in our ^18^F-FDG-based K_1_ analyses[42].
- k_3_ parameter represents the phosphorylation rate of ^18^F-FDG by hexokinase (HK) enzymes, which play a key role in glucose metabolism and are involved in hyperactive cells, including immune and malignant disorders [43]. Recent region-based k_3_ analyses reported a 21-fold higher target-to-background ratio in tumors compared to SUV data [36].

Ones could argue the lack of robustness of kinetic parametric imaging, due to noise-related signal fitting inconsistencies. However, SUV data are also prone to signal variabilities, as we showed in our simulation study. Importantly, the numerous drawbacks reported for SUV over the past 30 years – including patient preparation, acquisition protocol, reconstruction parameters, normalization factors – did not prevent SUV-based semi-quantitative metrics to become the rule in PET imaging practice [44]. Despite numerous biases, and beyond ease of calculation, one key point for its clinical success is its high reliability, suitable for disease assessment [45]. Very recently, Patlak parametric imaging was reported to be as reliable as SUV-based imaging, highlighting its suitability for disease monitoring [46]. One major limitation to full kinetic modeling adoption is the long acquisition time (30–60 min). Shortening scan duration is therefore an active research area, with promising approaches including:

- Population-based input functions [35, 37, 47, 48]
- Scaling factors with deep-kernel noise reduction, potentially reducing acquisition time to 10–20 min for Patlak imaging [49]

Alongside these acquisition optimizations, the availability of fast, multi-manufacturer, and user-friendly kinetic modeling tools is crucial for large-scale validation of 4D-PET parametric biomarkers—which motivated the development of PET KinetiX. It is important to note that PET KinetiX employs an indirect-based kinetic modeling approach. While direct parametric imaging—where the model is integrated into the tomographic reconstruction—offers reduced noise, lower bias, and enhanced image contrast [33] it has limitations:

- Model mismatch artifacts can propagate across neighboring voxels, whereas indirect methods limit errors to the affected voxel only.
- Direct methods require raw data and correction term access, whereas PET KinetiX is designed for ease of use and independence from PET manufacturers.
- Direct reconstruction is feasible only for simplified models (e.g., Patlak), whereas PET KinetiX supports both simplified and advanced 2 TCM models, making it a more flexible tool for research and clinical applications.

The present study has several limitations. First, the simulations were generated without respiratory motion. Second, our simulations only included 2 iterations schemes (N = 2 and 6 for low noise and high spatial resolution respectively) reflecting clinical standard conditions of the hardware used. While this is the standard model for ^18^F-FDG, we are currently implementing k_4_ modeling (reversible 2 TCM) for broader applicability. Additional refinements, such as radiotracer time-spreading corrections, are also under development, which will be especially relevant for whole-body LAFOV PET imaging [50]. In the era of precision medicine, 4D-PET imaging is rapidly evolving. Recent pioneering studies have highlighted the need to rethink kinetic models from a whole-body perspective [51]. PET KinetiX was designed as a flexible solution, capable of adapting to future innovations in this field.

## Conclusion

PET KinetiX generates K_i_ and v_b_ parametric maps with qualitative rendering comparable to unprocessed SUV data, while improving CNR in most cases. The 2 TCM k_3_ parametric maps exhibited the highest CNR improvements for tumor structures, making them promising candidates for further applications in various anatomical regions and radiotracer studies.

## Supplementary Information

Below is the link to the electronic supplementary material.

Supplementary file1 (PNG 4205 KB)

## Data Availability

The datasets generated during and/or analysed during the current study are available from the corresponding author on reasonable request.
