# Supplementary figures and images for: Fast 4D-PET parametric imaging computation at the whole field of view level: Reliability under simulated conditions of PET KinetiX, a dedicated software solution
Source: Eur J Nucl Med Mol Imaging. 2025 Apr 21;52(11):4065–77. doi: 10.1007/s00259-025-07285-0 (PMC12397132; doi:10.1007/s00259-025-07285-0)

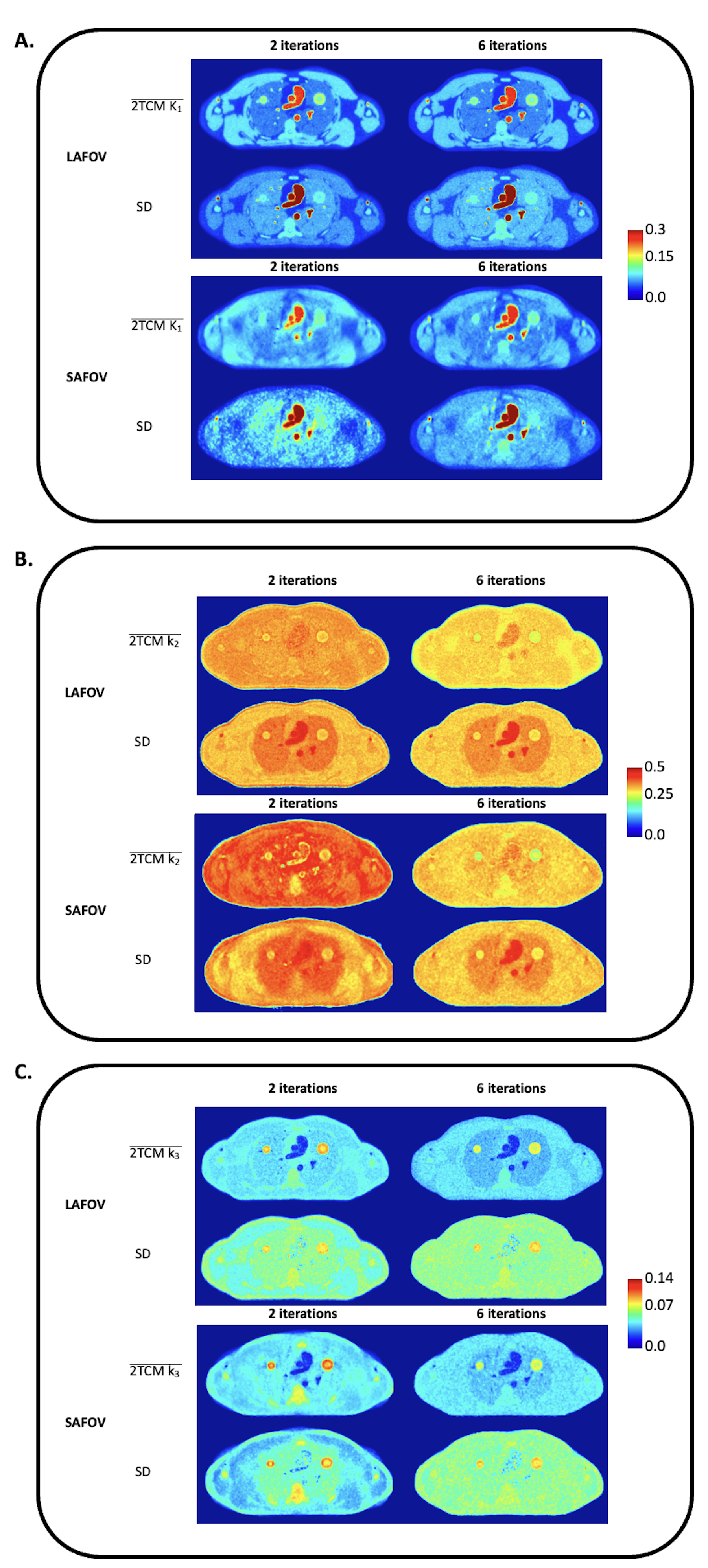

Supplement: Supplementary file 1 — Supplementary file1 (PNG 4205 KB) [file 259_2025_7285_MOESM1_ESM.png]
